# Transdiagnostic and Disorder-Specific Resting-State Functional Network Alterations in Alcohol Use Disorder, Schizophrenia, Bipolar Affective Disorder and Obsessive-Compulsive Disorder

**DOI:** 10.1192/j.eurpsy.2023.342

**Published:** 2023-07-19

**Authors:** P. A. Khadse, B. Holla, V. G, J. P. John, V. Benegal

**Affiliations:** 1 Department of Psychiatry, Jawaharlal Nehru Medical College, Datta Meghe Institute of Higher Education and Research, Wardha; 2 Department of Psychiatry, National Institute of Mental Health and Neurosciences, Bangalore, India

## Abstract

**Introduction:**

The ICD and DSM diagnostic categories do not represent entirely distinct entities because several cognitive impairments are shared across psychiatric disorders. Such shared cognitive impairments are hypothesized to be caused by common neurobiological substrates, one of which is transdiagnostic alterations in functional network connectivity (FNC).

**Objectives:**

To investigate and compare the within-network functional connectivity (WNFC) and between-network functional connectivity (BNFC) in alcohol use disorder (AUD), schizophrenia (SCZ), bipolar affective disorder (BPAD), obsessive-compulsive disorder (OCD) and healthy controls (HC) using resting-state fMRI employing a data-driven exploratory approach.

**Methods:**

The current study was a secondary analysis of data from the ADBS project in India. After pre-processing of fMRI data, a spatially and temporally constrained group-independent component analysis in the GIFT toolbox was performed using the NeuroMark templates to generate 53 independent components (ICs). These components were divided into seven functional domains including subcortical (SC), auditory (AU), sensorimotor (SM), visual (VI), cognitive-control (CC), default-mode (DM), and cerebellar (CB). To investigate the FNC correlations associated with group status (patients or HC) univariate models were applied which were subjected to corrections for multiple comparisons at an alpha=0.05 significance level using the FDR.

**Results:**

The overall sample size was 249 [AUD=35, SCZ=44, BPAD=48, OCD=53, and HC=69]. Transdiagnostic WNFC alterations largely involved dysconnectivity in the CC, DM, and SC domains, resulting in ICs with both increased and decreased WNFC. Transdiagnostic BNFC alterations were primarily in the form of increased connectivity of the SC domain with various cortical domains whereas reduced connectivity was noted between AU, VI, SM, and CB domains. There was AUD-specific hyperconnectivity in the CC domain and SCZ-specific hyperconnectivity in the DM domain, and dysconnectivity in the SC domain. BPAD-specific hyperconnectivity was identified in DM and SC domains in addition to increased connectivity between CB and SM domains and decreased connectivity between CB and SC domains. All results were significant at p ≤ 0.05; [FDR] q= 0.05.

**Conclusions:**

Our transdiagnostic WNFC alterations corresponded to the central executive network, default mode network, salience network, and CSTC loop, which provided transdiagnostic evidence for the triple network model of psychopathology and underlined the relevance of subcortical dysconnectivity in this model. Furthermore, our BNFC changes showed subcortical hyperconnectivity with many cortical networks, underscoring its relevance as a potential target for transdiagnostic therapeutic interventions.

**Disclosure of Interest:**

None Declared

